# Supplementary material for: Breaking the silence: Barriers to help-seeking among female victims of domestic violence in Ardabil, Iran – A qualitative study
Source: PLoS One. 2025 Jul 15;20(7):e0326998. doi: 10.1371/journal.pone.0326998 (PMC12262868; doi:10.1371/journal.pone.0326998)
Supplement: Attachment — Submitted filename: Response to Reviewer.docx [file pone.0326998.s001.docx]

**Dear Editor,**

We appreciate you and the reviewers for your precious time in reviewing our paper and providing valuable comments. It was your valuable and insightful comments that led to possible improvements in the current version. The authors have carefully considered the comments and tried our best to address every one of them. We hope the manuscript after careful revisions meet your high standards. The authors welcome further constructive comments if any.

Below we provide the point-by-point responses. All modifications in the manuscript have been highlighted in yellow.

Sincerely,

Zahra Behboodi Moghadam

Professor, School of Nursing & Midwifery, Tehran University of Medical Sciences, Tehran, Iran.

e-mail: Behboodi@tums.ac.ir

**Reviewer1:**

Comment 1. Title: Add a geographical or cultural indicator in the title to emphasize the study's regional and cultural relevance. For example, explicitly mentioning Iran or Ardabil province will highlight the contextual specificity of the research.

- Reply 1: Thanks for your kind reminders. This is corrected and highlighted in the manuscripts.
- Revised Title: *"Breaking the Silence: Barriers to Help-Seeking among Female Victims of Domestic Violence* *in Ardabil, Iran – A Qualitative Study"*

Comment 2. The study briefly discusses the necessity of "specialized services" and "financial and moral support" but does not propose innovative or unique solutions. To enhance its contribution, the authors should explore novel recommendations tailored to the Iranian context, particularly focusing on actionable strategies for systemic reform.

- Reply 2: Thanks for your kind reminders. This is corrected and highlighted in the manuscripts.

Contextual suggestions for problem-solving are also provided in discussion section:

-Creating community support centers for domestic violence victims: Establishing community support centers in Ardabil province that provide the necessary legal, psychological, and job training for women affected by domestic violence. They may be set up in conjunction with community health care centers to make it more convenient for people.

-Economic Empowerment Programs: Develop government-funded programs that provide women with access to microloans or grants to start microenterprises, thus reducing their economic dependence on their abuser.

-Cultural Awareness Initiatives: Work hand in hand with media campaigns and community and local faith leaders to culturally interrogate patriarchal norms and decrease the stigma around asking for help for domestic violence.

-Law Enforcement Practices: Ensure that police departments and the judicial system receive mandatory training and standards for responding to domestic violence and treatment of victims with respect and dignity

Comment 3. Results Section: While the results are well-structured into categories and subcategories, the explanations of these themes lack depth. Certain categories, such as "Unfavourable Socioeconomic Status" and "Lack of Women's Empowerment," overlap conceptually, which dilutes their distinctiveness. Consider merging overlapping categories or providing clearer distinctions.

- Reply 3: Thanks for your kind reminders. This is corrected and highlighted in the manuscripts.

 The categories "Unfavourable Socioeconomic Status" and "Lack of Women's Empowerment" merged into a single category named “**Structural and Economic Barriers to Empowerment**” with subcategories, including:

A) High costs of legal and social support services

B) Lack of economic independence for women

C) Lack of awareness of one’s rights

D) Lack of access to education and employment opportunities

E) Belief in women's vulnerability after divorce

Comment 4. Some subcategories are underdeveloped (e.g., "Lack of Economic Independence for Women"). These should be critically analyzed in more detail, with stronger contextualization in terms of societal and policy implications.

- Reply 4: Thanks for your kind reminders. This is corrected and highlighted in the manuscripts.

In Iran, many employers tend to prefer hiring men, and under equal conditions, women have fewer opportunities for employment. Additionally, in some regions of Iran, men prevent their wives and daughters from pursuing employment or continuing their education, which poses a significant barrier to women's financial independence.

Comment 5. The participant voices included are valuable, but the range of perspectives is limited. Ensure that diverse viewpoints, such as those of women from different age groups, education levels, and urban/rural settings, are adequately represented.

- Reply 5: Thanks for your kind reminders. In this study, purposive sampling with maximum variation was conducted, aiming to select women from diverse groups with different age ranges, educational backgrounds, and other characteristics.

Participants from diverse age groups (e.g., younger and older women), education levels (e.g., women with no formal education and those with advanced degrees), and urban/rural settings. This will provide a more comprehensive understanding of the barriers faced by women across different contexts.

- Younger women may face unique challenges related to societal expectations and lack of life experience.
- Women from rural areas may encounter additional barriers due to limited access to support services.
- Women with higher education levels may still face cultural stigma despite their educational attainment.

Comment 6. Novelty and Contextual Focus: The results do not highlight particularly unique or novel insights that distinguish this study from similar research in other settings. Emphasize findings that are specific to the cultural and social context of Iran to underscore the study's uniqueness. In both the results and discussion, ensure that claims about societal and structural barriers are explicitly linked back to the study's cultural and geographical context.

- Reply 6: Thanks for your kind reminders. This is corrected and highlighted in the manuscripts.

The cultural and social environment of Iran significantly influences women's experiences of domestic violence and their capacity to seek assistance. A more subtle cause is the patriarchal values embedded in our society which not only bolster male supremacy but also discourage women from reporting domestic abuse. Many women fear social stigma, family shame or a potential backlash from their abuser if they seek external help. Furthermore, women's rights and protection are restricted by legal and religious systems. Iran’s judicial system, informed by Islamic law, often favors men in family and legal disputes. For instance, a woman seeking a divorce due to domestic violence must provide substantial evidence, which is often difficult to obtain — and the methods for doing so are still extremely harsh. Other regulations, such as child custody and economic assistance laws, further discourage women from leaving abusive relations out of fear that they will lose their children or economic security. The other major factor is the stigma surrounding divorce, which has a significant impact on women's decisions to reach out for help. Divorced women often face social ostracism, decreased chances of remarriage and other opportunities, and impoverishment, which can create a reluctance to leave abusive marriages. Pressure to “protect the family” often supersedes personal health, compelling many women to remain in toxic situations.

Comment 7. The discussion makes broad claims without adequately emphasizing the study's specific contributions to the field of domestic violence research. Highlight how this study advances knowledge, particularly within reproductive health and gender-based violence in patriarchal societies.
While the authors propose general solutions (e.g., breaking cultural taboos, raising awareness), actionable and specific recommendations are lacking. For instance:
What specific policy reforms or legal changes are necessary in the Iranian context?
How can existing support systems (e.g., police, welfare organizations) be improved to address the barriers identified in the study?
Including a comparison with findings from other regions or countries with similar patriarchal norms could further enrich the discussion and provide valuable context.

Reply 7: Thanks for your kind reminders. This is corrected and highlighted in the manuscripts.

- Progress in Understanding Regarding Domestic Violence and Reproductive Health : We acknowledge the importance of demonstrating how this research contributes to the joint body of knowledge on domestic violence — particularly regarding reproductive health issues and gender-based violence in patriarchal societies. This research offers unique perspectives on how domestic violence is perpetuated in Iran by patriarchal standards, legal systems, and socio-cultural barriers. Focusing on women in this specific social and cultural context, we highlight the impact that legal and religious frameworks have on women's autonomy, access to a support system, and reproductive rights. Our findings add to the understanding of how social expectations, legal barriers, and economic dependence affect reproductive health and gender-based violence, issues that are not as well-studied in similar research.

The authors suggest these recommendations tailored to the Iranian context to support women against domestic violence:

- Creating community support centers for domestic violence victims: Establishing community support centers in Ardabil province that provide the necessary legal, psychological, and job training for women affected by domestic violence. They may be set up in conjunction with community health care centers to make it more convenient for people. Concerning the enhancement of current support systems, we suggest bolstering and broadening the functions of welfare organizations, shelters, and law enforcement agencies. In particular, we suggest that these organizations obtain increased financial support, specialized training regarding domestic violence, and enhanced legal protection to safeguard victims. A more organized strategy for coordination among the police, social services, and healthcare providers is crucial to guarantee that women receive the assistance they require at all phases

- Economic Empowerment Programs: Develop government-funded programs that provide women with access to microloans or grants to start microenterprises, thus reducing their economic dependence on their abuser.

- Cultural Awareness Initiatives: Work hand in hand with media campaigns and community and local faith leaders to culturally interrogate patriarchal norms and decrease the stigma around asking for help for domestic violence.

- Law Enforcement Practices: Ensure that police departments and the judicial system receive mandatory training and standards for responding to domestic violence and treatment of victims with respect and dignity

Comment 8. Conclusion: The conclusion reiterates points already made in the discussion and lacks a strong final message. Make it more concise and focused on the study's key takeaways, highlighting the most critical findings, their implications for reproductive health, and clear recommendations for policy and practice.

Reply 8: Thanks for your kind reminders. This is corrected and highlighted in the manuscripts.

Data analyses reveal the persistence of patriarchal norms in Iranian society and the systematic violations of legal rights, which constitutes the initial barrier for women when confronting domestic violence. This research shows that women find access to essential support services unattainable due to financial dependence on the abuser, oppressive legal frameworks, and the fear of social stigma. Moreover, both the legal system and social support structures fail them effectively, often leaving them trapped in abusive situations.

The high costs of legal processes and the long journey required to get satisfactory results from existing support organizations are significant challenges according to key findings. Economic and legal barriers prevent many women from reporting abuse or leaving their abusers. The stigma attached to divorce and the societal expectations of upholding family unity is another reason that prevents women from seeking help.

The study notes that such reforms will not be simple, but they are attainable. Iranian women can escape from domestic violence if such support systems are in place along with a supportive group of legal practitioners.

**Reviewer 2:**

The article is very significant and valuable and needs minor corrections.

Reply: thank you very much.

Comment 1. Abstract : Please write more important findings about age and education level in the results section.

Reply 1. Thanks for your kind reminders. This is corrected and highlighted in the manuscripts.

Background:
comment 2. Update all statistics provided in the background. 1993 is too old.

Reply 2. Thanks for your kind reminders. This is corrected and highlighted in the manuscripts.

Update Statistics: *Globally, 1 in 3 women (approximately 30%) experience physical or sexual violence in their lifetime, with intimate partner violence being the most common form (WHO, 2021). In Iran, recent studies indicate that domestic violence affects up to 66% of women, with significant regional variations (Alamolhoda et al., 2024).*

comment 3. The introduction is very long and lacks a clear procedure.

Reply 3. Thanks for your kind reminders. This is summarized.

comment 4. What is your novelty? Gap knowledge?

Reply 4. Thanks for your kind reminders. This is corrected and highlighted in the manuscripts.

While numerous studies have explored domestic violence globally, there is limited qualitative research on the barriers to help-seeking among women in Iran, particularly in regions like Ardabil province. This study addresses this gap by providing an in-depth understanding of the cultural, social, and structural barriers faced by Iranian women, offering context-specific insights that are crucial for developing effective interventions.

comment 5. Please summarize the study aim and add it to the last section of the background.

Reply 5. Thanks for your kind reminders. This is corrected and highlighted in the manuscripts.

This study aims to explore the barriers to help-seeking among female victims of domestic violence in Ardabil, Iran, with a focus on understanding the cultural, social, and structural factors that influence their decisions.

Methods:
comment 6. Lines 143: Maximum variation is correct

Reply 6. Thanks for your kind reminders. This is corrected and highlighted in the manuscripts.

comment 7. lines 144: Cite Table 1

Reply 7. Thanks for your kind reminders. This is corrected and highlighted in the manuscripts.

Results:
comment 8. Lines 196: First mention main categories and then sub-categories

Reply 8. Thanks for your kind reminders. This is corrected and highlighted in the manuscripts.

comment 9. In Table 2: Point out and provide some codes in Table 2 or the text.

Reply 9. Thanks for your kind reminders. We added some codes in Table 2.
