# Supplementary material for: Breaking the silence: Barriers to help-seeking among female victims of domestic violence in Ardabil, Iran – A qualitative study
Source: PLoS One. 2025 Jul 15;20(7):e0326998. doi: 10.1371/journal.pone.0326998 (PMC12262868; doi:10.1371/journal.pone.0326998)
Supplement: Attachment — Submitted filename: Response to Reviewers.docx [file pone.0326998.s002.docx]

**Dear Editor,**

We appreciate you and the reviewers for your precious time in reviewing our paper and providing valuable comments. It was your valuable and insightful comments that led to possible improvements in the current version. The authors have carefully considered the comments and tried our best to address every one of them. We hope the manuscript after careful revisions meet your high standards. The authors welcome further constructive comments if any.

Below we provide the point-by-point responses. All modifications in the manuscript have been highlighted in yellow.

Sincerely,

Zahra Behboodi Moghadam

Professor, School of Nursing & Midwifery, Tehran University of Medical Sciences, Tehran, Iran.

e-mail: Behboodi@tums.ac.ir

**Reviewer Comment 1:**

***This is an important study that will undoubtedly add to knowledge about Iranian women’s experiences of IPV. Methods of data collection and analysis are appropriate and well-described.***

Response:

Thank you very much for your positive and encouraging feedback. we sincerely appreciate your kind words and are glad to hear that you found the study valuable and the methods well-described. Your support is truly appreciated.

## Reviewer Comment 2:

Is “preferred silence” correct, according to the cited study, or is it that these women did not seek help? (Perhaps because of fear or a lack of avenues to do so.)

### Response:

Thank you for your insightful comment. You are correct in noting the distinction. The phrase “Preferred silence” may unintentionally imply a conscious or voluntary choice, whereas the cited study suggests that the women did not seek help often due to fear, lack of support systems, or societal pressures. To reflect this more accurately, we have revised the text to clarify that their silence was not necessarily a preference but rather a consequence of limited options and external constraints.
(See Page 5, Line 81)

## Reviewer Comment 3:

I tend to think that the term “exposed” sounds like it could be witnessing; “experiencing” clarifies that they were direct victims of violence.

### Response:

Thank you for your insightful comment. We agree that the term "experiencing" is more accurate in this context, as it emphasizes the participants' direct victimization rather than passive observation. We have replaced “exposed” with “experiencing” throughout the manuscript.
(See Page 6, Line 94)

## Reviewer Comment 4:

unhelpful responses from service providers?

### Response:

Thank you for pointing this out. We have clarified this sentence to specify the types of unhelpful responses reported by participants. The revised version reads:
“Staying with an abusive partner is sometimes facilitated by unhelpful responses from service providers, such as being dismissed, blamed, or advised to remain silent for the sake of family unity.”
(See Page 6, Line99)

**Results:**

## Reviewer Comment 5:

Provides a useful overview of the data (categories, subcategories, and codes). Excellent inclusion of relevant quotes. Recommendations (such as information, education, and opportunities for financial independence) are directly tied to the study’s findings.

### Response:

## Thank you for your positive and encouraging feedback. We appreciate your recognition of the clarity in Table 2 and the relevance of the quotes included. We are also glad that the linkage between the study’s findings and the recommendations was clear and meaningful. Your comments are greatly motivating and reinforce the direction of this research.

## Reviewer Comment 6:

find the subtitle of “Unprincipled Family Support” to be a bit unclear—perhaps “Lack of Family Support” or “Unsupportive Responses from Extended Family Members?”

### Response:

Thank you for this helpful suggestion. We agree that “Unprincipled Family Support” may be unclear or misleading. We have revised the subtitle to “Unsupportive Responses from Extended Family Members” throughout the manuscript to better reflect the intended meaning.

(See Page 13, 17, 18, Line 271, 292)

## Reviewer Comment 7:

Lines 335-338 could be clarified (regarding custody laws and how care of children after divorce may not be awarded to women).

### Response:

Thank you for pointing this out. We have revised the relevant section to provide clearer information about custody laws in Iran, highlighting that after divorce, “custody laws, along with the fear that custody arrangements may not be in their favor and that their children might be taken away from them, discourage many women from seeking help for domestic violence, because under Iranian custody laws, after divorce, custody is often awarded to the father or paternal relatives rather than the mother, which creates additional challenges for women.”

(See Page 21, Line 338)

**Discussion:**

**Reviewer Comment 8:**

***Excellent points regarding patriarchal societies and the imposition shame and stigma on victims rather than on those who use violence.***

***Important points regarding Iran’s judicial system and Islamic law.***

Response:

Thank you for your thoughtful comments on the discussion of patriarchal structures and the legal context in Iran**.** We are pleased that the analysis of the social stigma surrounding victims and the critical examination of the judicial and religious frameworks were found to be meaningful. These aspects were central to the study, and your recognition of their importance is truly appreciated.

## Reviewer Comment 9:

Regarding line 372: “must provide substantial evidence, which is often difficult to obtain — and the methods for doing so are still extremely harsh,” could some of the method be provided with e.g. in brackets?

### Response:

Thank you for your suggestion. We have revised the sentence to include examples of such methods. The updated sentence reads:
“A woman seeking a divorce due to domestic violence, must provide substantial evidence, which is often difficult to obtain such as medical certificates, police reports, or witness testimonies and the methods for doing so are still extremely harsh.”
(See Page 22, Line374)

## Reviewer Comment 10:

Regarding line 409: “Women who are victims of violence face economic problems and expect the government, healthcare systems, or social workers to help them in this regard.” Is this a realistic expectation? Are these services and systems available to help victims of IPV with financial/economic challenges?

### Response:

Thank you for this important comment. We recognize that although such expectations are common among victims, support systems in Iran, particularly in smaller cities like Ardabil, are often limited or unavailable. We have revised the sentence to reflect this reality more accurately:
“Although women who experience violence often face serious economic hardship and may expect support from government institutions, healthcare systems, or social workers, in reality, such support is often scarce, inaccessible, or insufficient.”
(See Page 24, Line412)

## Reviewer Comment 11:

While I appreciate that translation and understanding women’s experiences when translated to English from their original language could be a limitation, it is a strength of this study that it makes this information available to readers in English.

Thank you to the authors for their work on this important topic.

Response:

Thank you for highlighting the value of contextualized recommendations and the effort to make participants’ voices accessible in English. We truly appreciate your recognition of the challenges and importance of translation in qualitative research. It was our intention to preserve the authenticity of women’s experiences while making them understandable and meaningful to an international audience.

We appreciate the constructive feedback and the opportunity to improve our manuscript. We believe the revisions have strengthened the clarity and rigor of our study, and we thank the reviewers and editor again for their time and expertise.
